# Fyn phosphorylates AMPK to inhibit AMPK activity and AMP-dependent activation of autophagy

**DOI:** 10.18632/oncotarget.11916

**Published:** 2016-09-08

**Authors:** Eijiro Yamada, Shuichi Okada, Claire C. Bastie, Manu Vatish, Yasuyo Nakajima, Ryo Shibusawa, Atsushi Ozawa, Jeffrey E. Pessin, Masanobu Yamada

**Affiliations:** ^1^ Department of Medicine and Molecular Science, Gunma University Graduate School of Medicine, Maebashi, Japan; ^2^ Division of Biomedical Sciences, Warwick Medical School, Coventry, West Midlands, United Kingdom; ^3^ Departments of Medicine and Molecular Pharmacology, Albert Einstein College of Medicine, Bronx, New York, United States of America; ^4^ Nuffield Department of Obstetrics & Gynaecology, University of Oxford, Oxford, United Kingdom

**Keywords:** Fyn, AMPK, TNFα, Autophagy

## Abstract

We previously demonstrated that proto-oncogene Fyn decreased energy expenditure and increased metabolic phenotypes. Also Fyn decreased autophagy-mediated muscle mass by directly inhibiting LKB1 and stimulating STAT3 activities, respectively. AMPK, a downstream target of LKB1, was recently identified as a key molecule controlling autophagy. Here we identified that Fyn phosphorylates the α subunit of AMPK on Y436 and inhibits AMPK enzymatic activity without altering the assembly state of the AMPK heterotrimeric complex. As pro-inflammatory mediators are reported modulators of the autophagy processes, treatment with the pro-inflammatory cytokine TNFα resulted in 1) increased Fyn activity 2) stimulated Fyn-dependent AMPKα tyrosine phosphorylation and 3) decreased AICAR-dependent AMPK activation. Importantly, TNFα induced inhibition of autophagy was not observed when AMPKα was mutated on Y436. 4) These data demonstrate that Fyn plays an important role in relaying the effects of TNFα on autophagy and apoptosis via phosphorylation and inhibition of AMPK.

## INTRODUCTION

Although autophagy is originally described as an evolutionarily conserved cellular recycling program [1, 2], recent studies have demonstrated the role of autophagy in metabolic functions. Indeed autophagy participates in hepatic lipid storage [3], glucose homeostasis [4, 5], adipocyte differentiation [6, 7] and protects α cells against chronic lipid stress [8, 9]. Interestingly, deregulation of these functions is also associated with inflammatory processes, suggesting that inflammatory molecules modulate the autophagy function. In line with this, prolonged exposure of tissues and organs to high concentrations of inflammatory mediators inhibits autophagy response, resulting in severe cell damage and apoptosis [10, 11].

The molecular mechanisms underlining the cross talk between autophagy processes, energy metabolism and immune function have been under scrutiny. Recently, molecules traditionally known for their role in metabolism and homeostasis have been identified as elements of the autophagy pathway. Amongst them is the AMP-dependent protein kinase [AMPK], one of the major energy sensors of the cell [1, 12, 13]. AMPK is a highly conserved heterotrimeric complex composed of three subunits α, β and γ [14–16]. AMP binds to the γ subunit and increases the phosphorylation of the catalytic α subunit T172 residue by upstream kinases such as Ca^2+/^calmodulin-dependent protein kinase kinase (CaMKK2) in the brain and Liver Kinase B1 (LKB1) in peripheral organs [17, 18], resulting in the activation of AMPK kinase. The relative expression levels, association, and subcellular distribution of these upstream kinases in addition to the expression levels of the various AMPK α, β, γ subunit isoforms are responsible for the large pleiotropic responses of AMPK under a variety of cellular states [19].

Although the link between AMPK and autophagy is established, upstream regulators of AMPK in the context of autophagy response are still largely unknown. Previously, we reported that Fyn knockout (FynKO) mice display increased AMPK activity as a result of Fyn-dependent LKB1 regulation in peripheral tissues [20, 21]. Another recent publication also demonstrates that Fyn exerts its inhibitory effect on AMPK through both LKB1 and PIKE-A pathways [22]. Fyn is a member of the Src family of proto-oncogene non-receptor tyrosine kinases with multiple functions including the regulation of several inflammatory processes [23, 24]. Two major spliced isoforms, FynB and FynT have been reported [25], differing by their kinase activity (Fyn T being the most active isoform) and functions [26]. Interestingly, using muscle specific FynB or FynT over-expressing animals we recently demonstrated Fyn-dependent inhibition of autophagy via STAT3 regulation in muscle [27]. Although this pathway accounts for the ability of Fyn to suppress autophagy under basal conditions, other signalling events are likely to be involved under pathological conditions, particularly under metabolic stress, a condition associated with increased inflammatory processes and production of metabolic cytokines such as TNFα [28]. Interestingly, studies show that TNFα inhibits autophagy processes [10, 29] and others suggest that TNFα regulates AMPK activity [28, 30–32]. Yet, it has not been fully examined whether TNFα inhibits autophagy through AMPK.

In this study, we demonstrate that 1) Fyn specifically phosphorylates AMPKα on Y436, resulting in AMPK activity inhibition and thereby suppressing autophagy, 2) TNFα activates Fyn kinase, 3) Fyn deficiency blocks TNFα inhibition of autophagy by preventing AMPK α subunit Y436 phosphorylation and 4) Fyn deficiency protects against TNFα activation of apoptosis.

## RESULTS

### AMPK undergoes Fyn-dependent tyrosine phosphorylation

Having recently identified LKB1 and STAT3 as direct substrates of Fyn in the respective regulation of metabolism [21] and muscle autophagy [27], we wished to identify other potential Fyn targets. Skeletal muscle extracts from Fyn over-expressing and FynKO mice were subjected to mass spectrometry analysis [27]. Along with the increased STAT3 Y705 phosphorylation in Fyn over-expressing mice, we also found a marked increase in phosphorylation on Y436 of the AMPK α subunit (Supplementary Figure 1A, 1B), suggesting that Y436 could be a specific site for Fyn.

**Figure 1:**
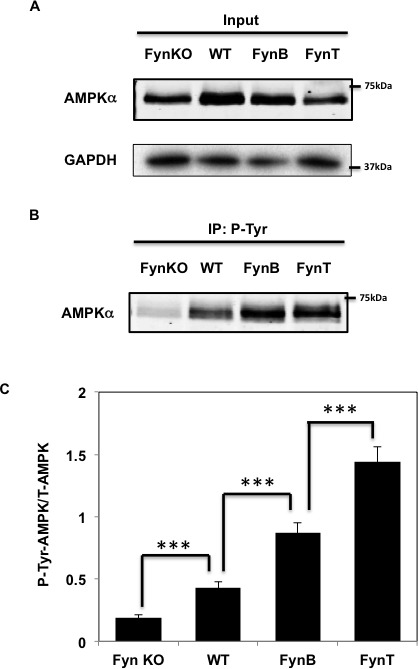
Fyn phosphorylates AMPKα in skeletal muscle **A.** Whole cell lysates from gastrocnemius muscle of either FynKO mice, WT, HSA-FynB (FynB) or HSA-FynT (FynT) mice were homogenized and immnoblots were performed by AMPKα mAb and GAPDH **B.** Gastrocnemius muscle of either FynKO mice, WT, HSA-FynB (FynB) or HSA-FynT (FynT) mice were homogenized and immunoprecipitated with 4G10 mAb followed by immunoblotting with AMPK mAb. Blots are representative of 3 independent experiments. **C.** Signal quantification of tyrosine-phosphorylated AMPKα levels from (A) and (B). Statistical examination between each set was performed. ***P < 0.005.

To confirm Fyn-dependent *tyrosine* phosphorylation of AMPK, gastrocnemius muscle tissue extracts were prepared from Fyn knockout (FynKO), wild type (WT), skeletal muscle-specific over expressing FynB and FynT mice. The extracts were immunoblotted for AMPK α subunit and GADPH as a loading control (Figure 1A). Immunoprecipitation of these extracts with the 4G10 phospho-tyrosine specific antibody demonstrated a low level of AMPK *tyrosine* phosphorylation in the FynKO mice compared to control WT mice (Figure 1B). In contrast, skeletal muscle extracts from the FynB and FynT overexpressing mice displayed an approximate 2-fold increase in AMPK α subunit *tyrosine* phosphorylation compared to wild type mice. FynT is the most active isoform of Fyn, yet AMPKα phosphorylation levels in FynT mice were similar to those seen in FynB mice. However, we found that total AMPKα expression (Figure 1A) in FynT muscle was decreased compared to WT, FynKO or FynB mice, likely due to the fact that FynT mice display a strong sarcopenic phenotype [27] known to decrease AMPKα expression [28, 33]. Therefore, when normalized to total AMPKα, protein levels, *tyrosine* phosphorylation of AMPKα in FynT mice was further increased when compared to FynB mice (Figure 1C).

**Figure 2:**
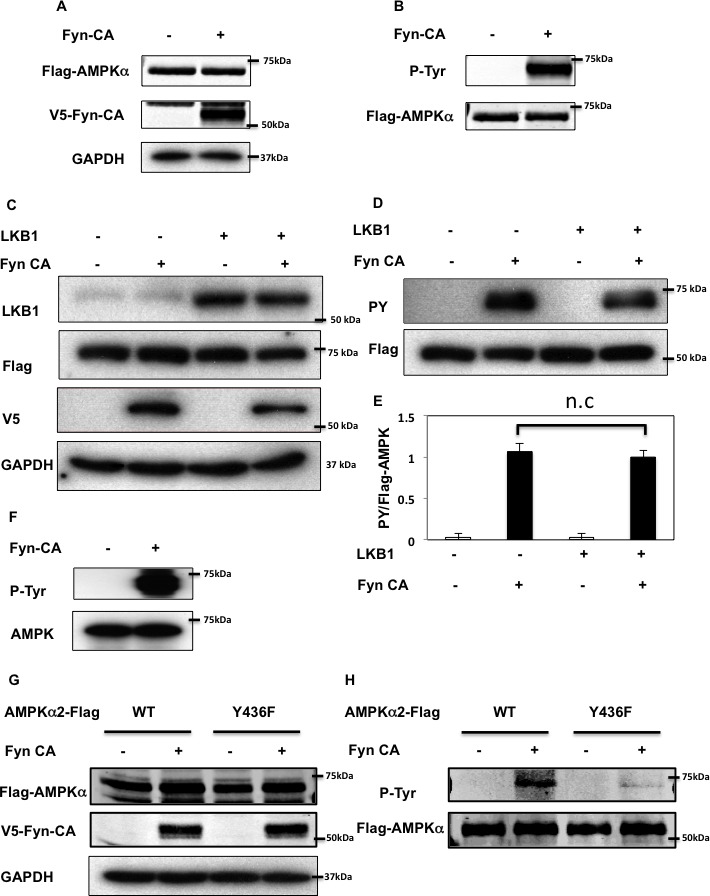
Fyn phosphorylates AMPK on tyrosine 436 **A., B.** NIH3T3 cells were co-transfected with 3myc-AMPKα2-Flag and Fyn-CA-V5. 48 h later, cells extracts were prepared and immunoprecipitated with a Flag antibody. Immunoblots of lysates (A) and immunoprecipitation samples (B) were performed with the indicated antibodies. Blots are representative of 3 independent experiments. **C., D.** Forty-eight hours after co-transfection with 3myc-AMPKα2-Flag, Fyn-CA-V5 and pcDNA3-LKB1, HEK293T cells extracts were prepared and immunoprecipitation was performed using a Flag antibody. Immunoblots of lysates (C) and immunoprecipitation samples (D) were performed with the indicated antibodies. These are representative immunoblots independently performed 3 times. **E.** Signal quantification of tyrosine-phosphorylated AMPKα levels from (D). Statistical examination between each set was performed. **F.** Purified Human PRKAA2 (100 ng) and either mock or Flag-Fyn-CA (380 ng) were incubated with the kinase buffer and ATP at 30°C for 30 min. Samples were boiled and separated onto a 8% SDS-PAGE gel, followed by immunoblotting with the indicated antibodies. **G., H.** HeLa cells were co-transfected by either 3myc-AMPKα2 WT or Y436F (YF) with Fyn-CA-V5. Immunoprecipitation was performed using a Flag antibody. Immunoblots of lysates (G) and immunoprecipitation samples (H) were performed with the indicated antibodies. Images are representative of 4 independent experiments.

To determine if Fyn phosphorylates the AMPKα in cell culture conditions, we co-expressed an epitope tagged AMPKα2 (AMPKα2-Flag) cDNA with or without an epitope tagged constitutively active Fyn (V5-Fyn-CA) cDNA in NIH-3T3 cells (Figure 2A). Expression of V5-Fyn-CA resulted in the robust *tyrosine* phosphorylation levels of AMPKα2-Flag (Figure 2B). We recently reported that Fyn directly phosphorylates LKB1 to modulate AMPK activity [21]. To evaluate whether AMPK *tyrosine* phosphorylation is also dependent on LKB1, we co-expressed LKB1 in HEK293T cells. LKB1 had no effect on V5-Fyn-CA or AMPKα2-Flag expression. Importantly, *tyrosine* phosphorylation of AMPKα2-Flag in presence of Fyn was unaffected by LKB1 co-expression (Figure 2C, 2D, 2E). These data demonstrate that Fyn-dependent *tyrosine* phosphorylation of AMPKα is independent of LKB1.

To demonstrate that AMPKα is a direct Fyn substrate, we incubated human purified AMPKα2 protein with purified Fyn-CA (Figure 2F). In the presence of ATP, Fyn was observed to robustly phosphorylate the AMPKα2 subunit. The specificity for Y436 was confirmed by expression of the Y436F AMPKα mutant (Y436F-AMPKα2-Flag) with V5-FynCA in HeLa cells. As readily apparent in Figure 2G and 2H, the ability of V5-Fyn-CA to tyrosine phosphorylate the Y436F AMPKα2-Flag mutant was reduced compared to WT AMPKα2-Flag, demonstrating that Fyn specifically phosphorylates AMPKα2 on Y436.

### Tyrosine phosphorylation of the AMPK α2 subunit residue Y436 inhibits AMPK activity

**Figure 3:**
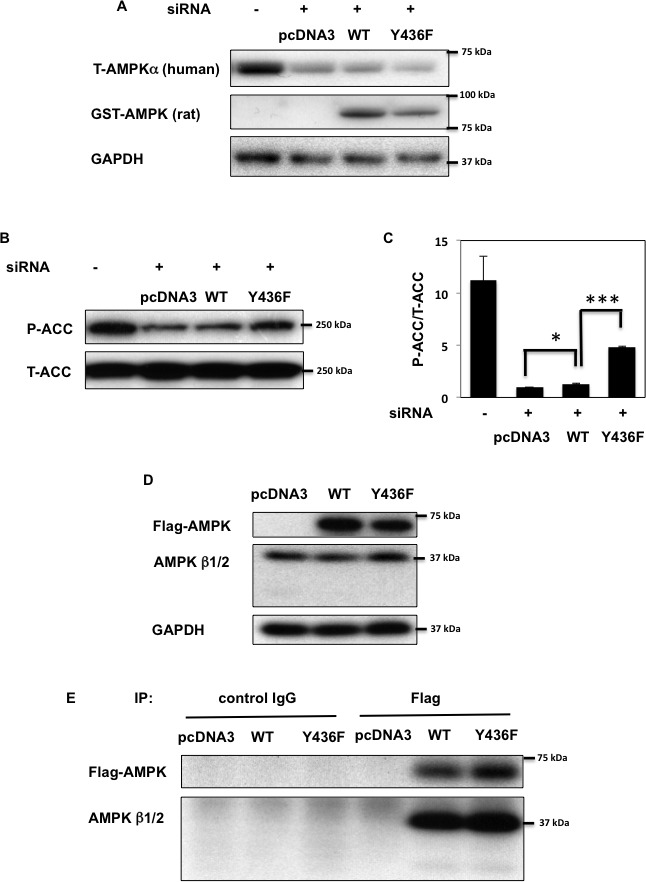
AMPK activity is decreased by tyrosine phosphorylation **A.** HEK293T cells were co-transfected with human siAMPK α1 and α2 subunits and either empty vector (pcDNA3), GST-rat AMPKα WT (WT) or Y430F mutant (equivalent to human Y436F (Y436F)) that are not affected by human AMPKa siRNA. 72 h after transfection immunoblots were performed with the indicated antibodies. **B., C.** S79 phosphorylation of ACC and total ACC levels in samples from (A) were assessed (B) and signal quantification of the expression levels of S79 phosphorylation of ACC normalized with total ACC were performed (C). Statistical examination between each sets were performed *p < 0.05, ***p < 0.005. **D.**, **E**. HEK293T cells were transfected by either 3myc-AMPKα2 WT or Y436F-Flag (Y436F). Co-immunoprecipitation was performed by either control IgG or AMPK β1/β2 polyclonal antibody. Immunoblots of lysates (D) and immunoprecipitation samples (E) were performed with the indicated antibodies. Images are representative of 3 independent experiments.

To evaluate whether tyrosine phosphorylation on Y436 has an effect on AMPK activity, we silenced the endogenous AMPK α1 and α2 subunits in HEK293T cells using specific human siRNA. As shown in Figure 3A, siRNA-mediated knockdown of AMPKα greatly reduced endogenous AMPKα protein expression levels. The knockdown cells were then transfected with an empty vector (pcDNA3), rat GST-AMPKα1-WT (WT) or the GST-AMPKα1-Y430F mutant (the equivalent to human Y436 of AMPK α2 subunit (Y436F)). We selected these rat constructs as they are not affected by human AMPKα siRNA and therefore continue to be expressed in HEK293T cells transfected by human AMPKα siRNA. Phosphorylation of acetyl-CoA carboxylase (ACC) on S79 (pS79-ACC), the specific AMPK site on ACC [34, 35] was used as a read-out for AMPK activity. As apparent in Figure 3B and 3C, siRNA mediated knockdown of endogenous AMPK reduced the basal level of pS79-ACC. Re-expression of rat GST-AMPKα1-WT resulted in a relatively small but significant increase in pS79-ACC whereas re-expression of rat GST-AMPKα1-Y430F increased ACC phosphorylation to a greater extent. The relative weak effect of rat GST-AMPKα1-WT to reconstitute AMPK dependent ACC phosphorylation was likely due to either the use of rat GST-AMPKα1 fusion protein or it might be that the rat protein does not fully compliment the human α1 subunit in HEK293T cells. Nevertheless, these data demonstrate that the Y436F AMPKα mutant displays significantly more AMPK activity than WT-AMPKα.

Recently, studies have demonstrated that AMPK kinase activity is regulated by modification of the AMPK α, β, γ subunit trimeric complex assembly state [14, 15, 17]. To determine whether Fyn suppresses AMPK activity through dissociation of the α, β, γ subunit interactions, we performed a series of co-immunoprecipitation experiments (Figure 3D, 3E and supplementary Figure 2A and 2B). Co-immunoprecipitation analyses revealed that phosphorylation of AMPK Y436 had no effect on the assembly status of the AMPK trimeric complex, indicating that Fyn-dependent phosphorylation and inhibition of AMPK activity does not result from AMPK subunit dissociation.

### Prolonged TNFα treatment diminishes AICAR-dependent AMPK activation through Fyn

Although a number of studies have shown that the pro-inflammatory cytokine TNFα regulates AMPK activity [10, 29], the molecular mechanisms underlying this regulation are still unclear [10, 29, 31, 32]. To test the hypothesis that TNFα might regulate AMPK through Fyn-dependent phosphorylation, we first examined acute and prolonged effects of TNFα incubation on AMPK T172 phosphorylation levels.

**Figure 4:**
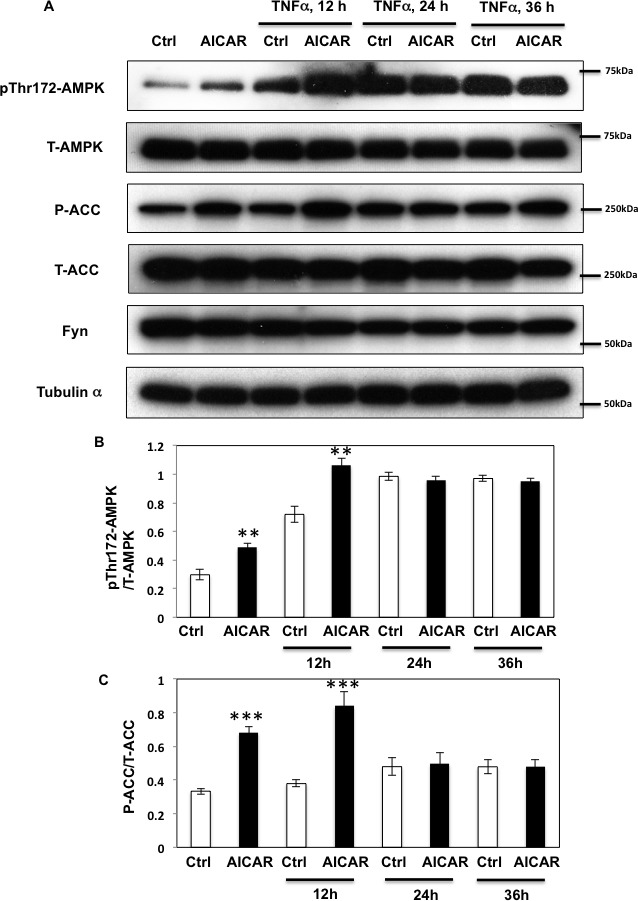
Prolonged TNFα stimulation suppresses AICAR dependent AMPK activation **A.** HEK293T cells were incubated with TNFα (10 ng/ml) for 12, 24 or 36 h and then stimulated with 2 mM AICAR for 10 min. Immunoblots were performed with the indicated antibodies. These are representative images from 3 independent experiments. **B., C.** Signal quantifications of expression levels of phospho- T172 AMPKα corrected by total AMPKα (B) and levels of phospho- S79 of ACC to total ACC (C). The data are presented as mean ± s.e.m. Statistical examination between control and AICAR stimulated cells in each sets were performed. **p < 0.01, ***p < 0.005.

**Figure 5:**
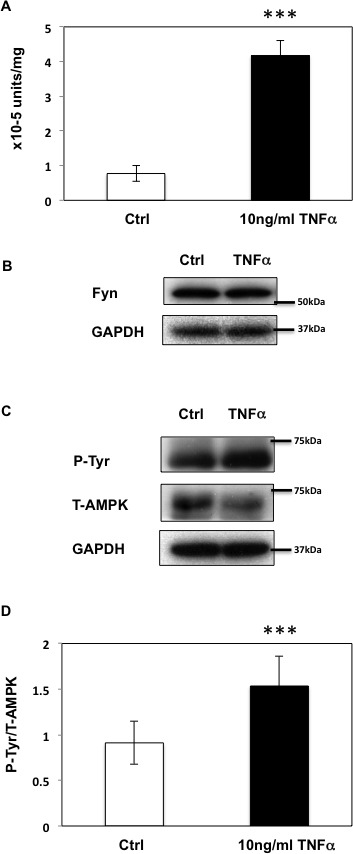
Prolonged TNFα stimulation up-regulates Fyn activity in HEK293T cells **A.** Fyn kinase activity in HEK293T cells treated or not with TNFα (10ng/ml) for 36 h. These are representative images from 5 independent experiments ***p < 0.005. **B.** Fyn expression in HEK293T cells treated or not with TNFα for 36 h. **C.** After a 36-h incubation with 10ng/ml TNFα, HEK293T cells were treated or not with TNFα for 36 h and homogenized. Immunoprecipitation was performed with total AMPKα antibody followed by immunoblotting with the 4G10 and total AMPKα antibody. These are representative images from 4 independent experiments. **D.** Signal quantification (Y436 normalized to total AMPKα) from (C). (***P < 0.005).

Short term (12 h) of TNFα treatment enhanced both basal and AICAR-stimulated T172 AMPKα phosphorylation levels (Figure 4A and 4B). In contrast, while prolonged incubation with TNFα (24-36 h) still enhanced T172 phosphorylation levels in basal condition, additional AICAR-dependent phosphorylation was abolished. Similarly, AICAR-dependent ACC phosphorylation was also further enhanced by TNFα treatment at 12 h but not at 24 or 36 h (Figure 4A and 4C). TNFα treatment also enhanced Fyn kinase activity in a cell extract assay (Figure 5A), without any significant change in Fyn protein levels (Figure 5B). Moreover this was associated with increased endogenous AMPKα subunit tyrosine phosphorylation (Figure 5C and 5D).

**Figure 6:**
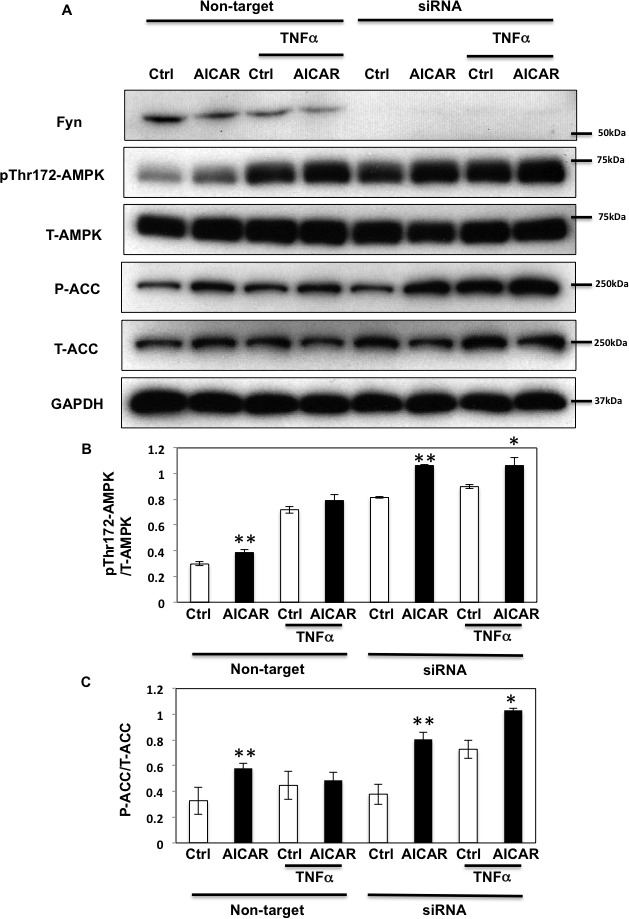
TNFα regulates AICAR-dependent AMPK activation via Fyn kinase **A.** HEK293T cells were transfected with Fyn siRNAs. Cells were treated with TNFα (10 ng/ml) for 36 h with or without 2 mM AICAR for 10 min. Immunoblots were performed with the indicated antibodies. These are representative images from 3 independent experiments. **B., C.** Signal quantifications of expression levels of phospho- T172 AMPKα normalized to total AMPKα (B) and levels of S79 phosphorylation of ACC normalized to total ACC (C). The data are presented as mean ± SE. Statistical examination between control and AICAR stimulated cells in each sets were performed. *P < 0.05, **P < 0.01.

To further investigate the role of Fyn in relaying the effects of TNFα on AMPKα phosphorylation, we next silenced Fyn protein expression by siRNA in HEK293T cells. As shown in Figure 6, AICAR increased AMPKα phosphorylation (Figure 6A and 6B) and subsequent ACC phosphorylation (Figure 6A and 6C) in non-target siRNA transfected HEK293T cells. Prolonged TNFα treatment increased the basal state of AMPKα phosphorylation but not that of ACC, suggesting that despite the increase of T172 phosphorylation, AMPK activity was not optimal. Additionally, neither AMPKα nor ACC phosphorylation levels were increased following AICAR stimulation, demonstrating that chronic TNFα blunts the effect of AICAR. However, AMPK and ACC phosphorylation levels were restored and increased in Fyn knockdown cells, suggesting that lack of Fyn the HEK293T cells prevented TNFα inhibition of AICAR-stimulated AMPKα T172 phosphorylation and activation.

### Fyn regulates autophagy through AMPK phosphorylation to regulate apoptosis

**Figure 7:**
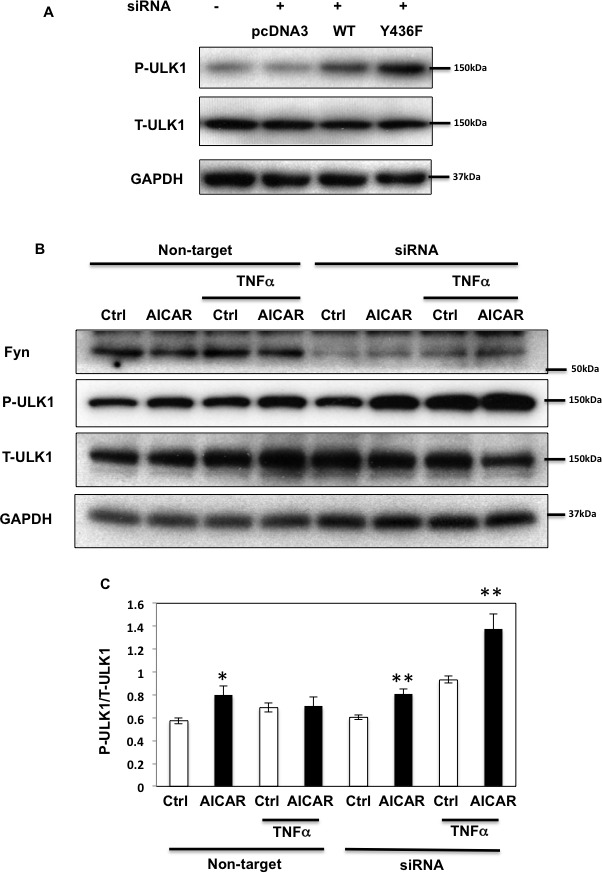
Fyn-mediated AMPK regulation inhibits ULK1 phosphorylation **A.** HEK293T cells were co-transfected with siRNAs of human siAMPK α1 and α2 subunits and either empty vector (pcDNA3), GST-rat AMPKα WT (WT) or Y430F mutant (equivalent to human Y436F (Y436F)) to be replaced. Immunoblots were performed 72 h after transfection. **B.** HEK293T cells were transfected with Fyn siRNAs. Cells were treated with 10ng/ml TNFα for 36 h with or without 2 mM AICAR for 10 min. Immunoblots were performed with the indicated antibodies. These are representative images from 3 independent experiments. **C.** Signal quantifications of the expression levels of phospho-S555 ULK1 to total ULK1. The data are presented as mean ± SE. Statistical examination between control and AICAR stimulated cells in each sets were performed *P < 0.05, **P < 0.01.

**Figure 8:**
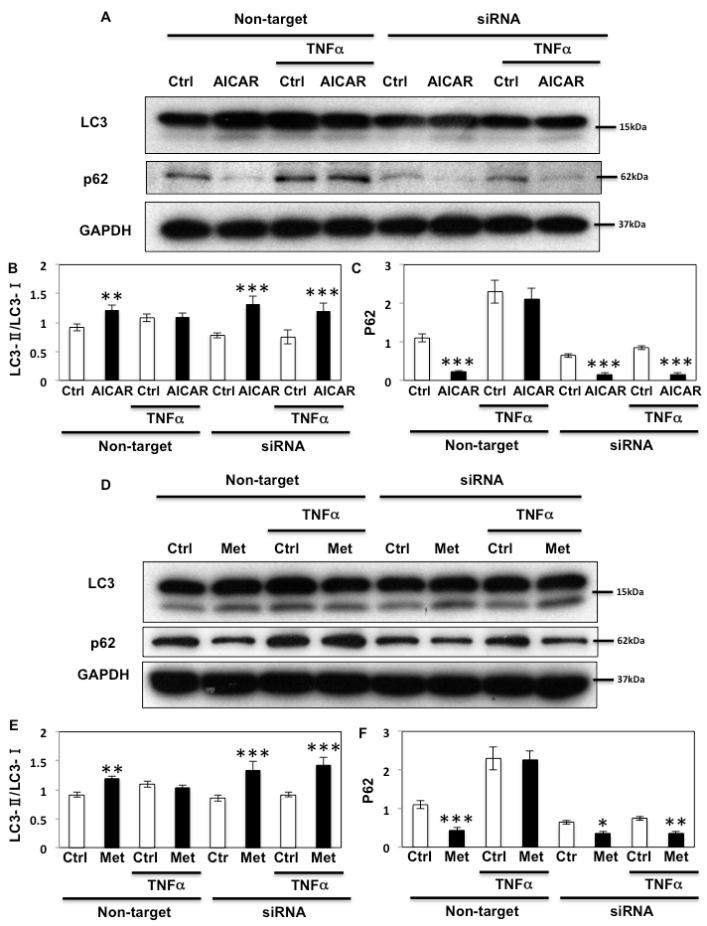
Fyn regulates metabolic stress- induced autophagy through AMPK **A.** HEK293T cells were transfected with siRNA for Fyn. The cells were treated with 10 ng/ml TNFα for 36 h with or without 2 mM AICAR for 10 min and immunoblots were performed with the indicated antibodies. These are representative images independently performed 3 times. **B., C.** Signal quantifications of the ratio of LC3-II to LC3-I (B) and p62/GAPDH (C). The data are presented as mean ± s.e.m. Statistical examination between control and AICAR stimulated cells in each sets were performed **P < 0.01, ***P < 0.005 **D.** HEK293T cells were transfected with siRNA for Fyn. Cells were treated with 10 ng/ml TNFα for 36 h with or without 5 mM Metformin for 20 min and immunoblots were performed with the indicated antibodies. These are representative images independently performed 3 times. **E., F.** Signal quantifications of the ratio of LC3-II to LC3-I (E) and p62/GAPDH (F). The data are presented as mean ± s.e.m. Statistical examination between control and AICAR stimulated cells in each sets were performed **P < 0.01, ***P < 0.005.

As AMPK plays an important regulatory function in the control of autophagy [1, 36], we next examined the role of Fyn-dependent AMPK regulation in this process. For this, HEK293T cells in which endogenous AMPK α1 and α2 subunits were silenced (as in Figure 3) were transfected with the empty vector (pcDNA3), GST-AMPKα-WT (WT) or the GST-AMPKα1-Y430F mutant (the equivalent to human Y436 of AMPK α2 subunit (Y436F)). ULK1 phosphorylation on the AMPK activating site was then quantified [12, 13]. GST-AMPK-WT expression increased ULK1 phosphorylation and expression of the rat GST-AMPK-Y430F mutant resulted in a further increase (Figure 7A). We next examined the effect of Fyn knockdown on ULK1 phosphorylation levels in basal, AICAR-stimulated and prolonged TNFα stimulated cells (Figure 7B and 7C). AICAR significantly increased ULK1 phosphorylation indicating that autophagy was up regulated, and TNFα blunted this effect. Importantly siRNA mediated knockdown of Fyn prevented the chronic TNFα inhibition of AICAR-stimulated ULK1 phosphorylation (Figure 7B and 7C). In line with this, AICAR increased the ratio of LC3-II/LC3-I and decreased expression of p62, consistent with an increase in autophagic activity (Figure 8A, 8B and 8C). While this was blunted when cells were treated with TNFα, siRNA-mediated knockdown of Fyn prevented this chronic TNFα inhibition of AICAR-stimulated autophagy activation. AICAR is an adenosine analog that directly modulates AMPK activity, and these results suggested the direct TNFα-Fyn regulation of the AMPK-autophagy axis. To confirm that Fyn mediates autophagy via AMPK we next took advantage of metformin, a known AMPK activator that is also an autophagy inducer. As shown Figure 8D, 8E and 8F, metformin increased the ratio of LC3-II/LC3-I and decreased p62 in the basal state, which is consistent with an increase in autophagic activity. Again, while this was blunted when cells were treated with TNFα, siRNA-mediated knockdown of Fyn prevented this chronic TNFα inhibition of metformin-stimulated autophagy activation (Figure 8D, 8E and 8F).

**Figure 9:**
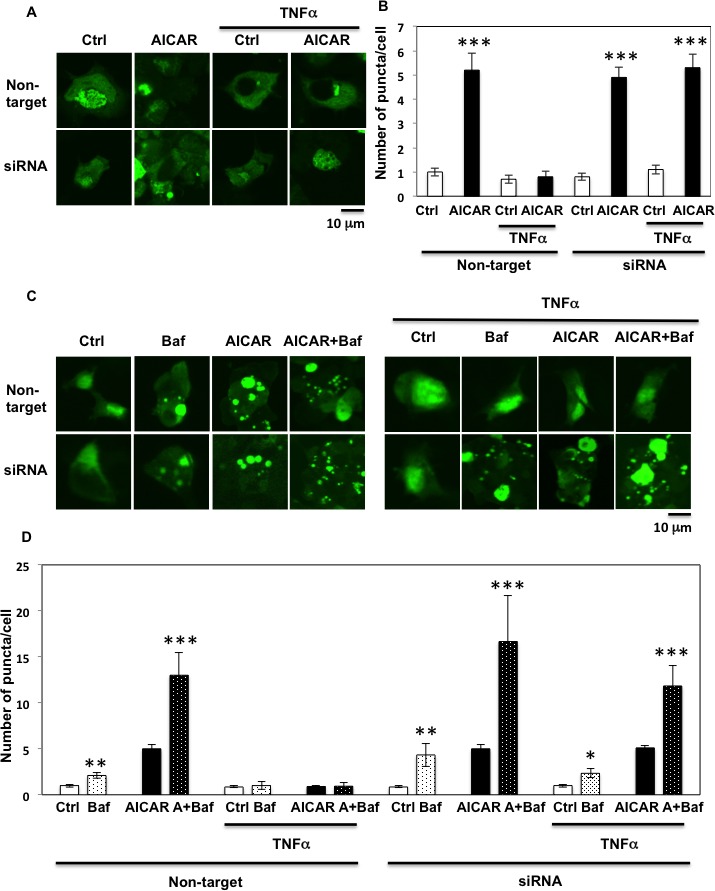
GFP-LC3 puncta formation assay confirms Fyn's regulation of AICAR dependent autophagy **A.** HEK293T cells were transfected with both siRNA for Fyn and GFP-human LC3 construct. The cells were treated with 10 ng/ml TNFα for 36 h with or without 2 mM AICAR for 10 min. Cells were fixed with 4% paraformaldehyde and mounted with DAKOCytomation Fluorescent Mounting Medium (S3023). These are representative images of high power field from experiments independently performed 3 times. **B.** Number of puncta in each single cell. Data are representative of *n* = 5 experiments. Statistical examination between control and AICAR-stimulated cells in each sets were performed ***P < 0.005. **C.** HEK293T cells were transfected with both siRNA for Fyn and GFP-human LC3 construct. Cells were treated with 10 ng/ml TNFα for 36 h with or without 100 nM Bafilomycin for 4 h and 2 mM AICAR for 10 min. Cells were fixed with 4% paraformaldehyde and mounted with DAKOCytomation Fluorescent Mounting Medium (S3023). These are representative images of high power field from experiments independently performed 3 times. **D.** Number of puncta in each single cell. Data are representative of *n* = 5 experiments. Statistical examination between control and AICAR stimulated cells in each sets were performed ***P < 0.005.

To confirm these results, we utilized the GFP-LC3 puncta formation assay. As shown Figure 9A, 9B and supplementary Figure 3A, AICAR increased the number of puncta in each cell, which is consistent with an increase in autophagic activity. While TNFα suppressed the AICAR induction of GFP-LC3 puncta, this was essentially completely reversed by siRNA-mediated knockdown of Fyn (Figure 9A, 9B and supplementary Figure 3A). While GFP-LC3 puncta formation assay indicates the levels of LC3 lipidation, it is not always correlated with the increase of autophagy flow. To confirm our results, we utilized the lysosomotrophic agent Bafilomycin A1 to inhibit the autophagy flow. As shown Figure 9C, 9D and supplementary Figure 3C and 3D, Bafilomycin A1 treatment significantly increased GFP-LC3 puncta formation in the basal state and AICAR accelerated this process, indicating that even in basal state the HEK293T cells have a certain level of autophagy flow that is increased by AICAR. Importantly Bafilomycin A1 was unable to increase GFP-LC3 puncta in the absence or presence of AICAR in TNFα-treated cells. In contrast, siRNA-mediated knockdown of Fyn restored the autophagy flow in the TNFα-treated cells both in the absence and presence of AICAR (Figure 9C, 9D and supplementary Figure 3B and 3C).

**Figure 10:**
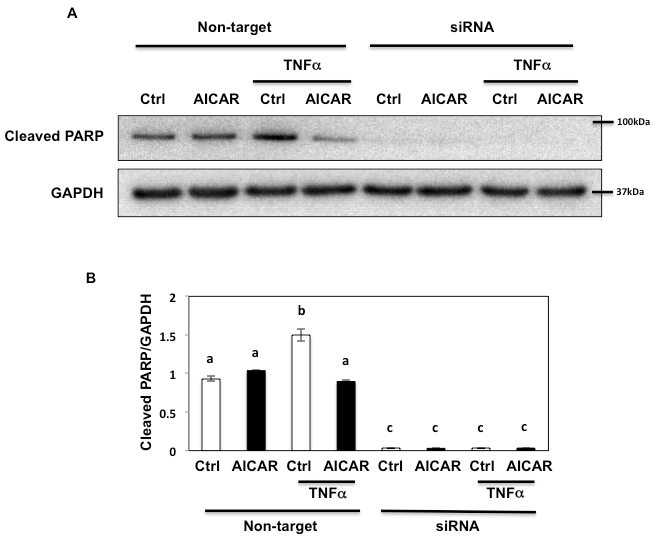
Fyn regulates metabolic stress- induced PARP cleavage through AMPK **A.** HEK293T cells were transfected with siRNA for Fyn and treated with 10 ng/ml TNFα for 36 h with or without 2 mM AICAR for 10 min. 150 μg of Lysate were immunoblotted by cleaved PARP pAb (#5625, Cell Signaling) and GAPDH mAb. These are representative images from experiments independently performed 3 times. **B.** Signal quantifications of cleaved PARP/GAPDH. The data are presented as mean ± s.e.m. Non-identical letters (a, b and c) indicate results that are statistically different from each other at *p* < 0.05.

Since prolonged exposure of inflammatory mediators inhibit autophagy resulting in severe cell damage and apoptosis [10], we next examined the role of Fyn in apoptosis. As shown Figure 10A and 10B, AICAR did not change the basal state level of apoptosis reflected by PARP cleavage. This lack of AICAR protection is likely due to a limited basal apoptotic response in HEK293T cells, a fact that we confirmed as we were unable to detect CHOP induction in these cells (not shown). However under these conditions, TNFα slightly induced PARP cleavage, which was rescued by AICAR, indicating that TNFα-induced apoptosis could be regulated by AMPK. Interestingly siRNA-mediated knockdown of Fyn resulted in a complete loss of PARP cleavage indicating that apoptosis was completely blocked (Figure 10A and 10B). These data indicate that TNFα-induced apoptosis might partially be regulated by AMPK through Fyn, although our data also suggest that Fyn regulates apoptosis independently of AMPK.

## DISCUSSION

AMPK is a master energy sensor in cells and it is activated in various conditions where cellular ATP is decreased. AMPK also has a major role in pathological conditions (e.g. metabolic syndrome, cancer, Alzheimer's disease) and therapeutic activation of AMPK is believed to have beneficial advantages for several disease processes. For example, metformin increases AMPK activity and is the most commonly prescribed oral anti-diabetic agent [34, 35] and recent studies have also shown that metformin therapy decreases cancer risk [37].

AMPK is a heterotrimeric complex composed of three different subunits α, β, γ and currently only two AMPK kinases (LKB1 and CaMKK2) and two AMPK phosphatases (PP2A and PP2C) are known to regulate AMPK activity [17, 18, 38, 39]. AMPK regulation is mainly affected by the tissue distribution of these upstream kinases. For example, LKB1 is expressed in peripheral tissues (liver, skeletal muscle and adipose) while CaMKK2 is present in neuronal cells [40, 41]. We identified one member of the Src family of proto-oncogene non-receptor tyrosine kinases, Fyn that regulates AMPK activity in peripheral tissues by directly interacting with LKB1 [21].

In this study, we now demonstrate that Fyn also directly regulates AMPK. We found that Fyn phosphorylates the AMPK α subunit on Y436 and this phosphorylation suppresses AMPK activity. The AMPK α subunit is composed of a kinase domain (KD) and a regulatory C-terminus. The regulatory C-terminus has an auto-inhibitory domain (AID), a sensor (αRIM) loop and a β-subunit interaction domain (β-ID). Additionally, a recent publication has implicated that AMP binding to CBS-3 of γ subunit induces the interaction of AID-αRIM2 and γ unit resulting in AMPK activation by releasing AID inhibition of the kinase domain [42]. Interestingly, inspection of AMPK sequence revealed that Y436 is localized in β-ID immediately after the αRIM-2 that is 2^nd^ part of αRIM and interacting with AID. This suggested that Y436 phosphorylation could affect AMPK subunits assembly. However, we found that Y436-dependent inhibition of AMPK activity was not due to the assembly state of the AMPK heterotrimeric complex (Figure 3C, 3D and supplementary Figure 2A, 2B) and since there is no change in the binding affinity between α subunit and β subunit by tyrosine phosphorylation of AMPK α subunit, we speculate that phosphorylation of Y436 may modify the AID-αRIM2 interaction.

Utilizing TNFα, a pro-inflammatory cytokine, we next examined the role of Fyn regulation of AMPK under metabolic stress conditions. Recent studies have reported that TNFα infusion into lean animals increases PP2C expression, resulting in a reduction in AMPK signaling, fatty acid oxidation, and the development of skeletal muscle insulin resistance [28, 30], but acute incubation of TNFα regulated AMPK activity due to a reduction in the AMP/ATP ratio at least in 3T3L1 adipocytes and in the epithelium-lymphocyte co-culture state [31, 32]. Therefore we examined the chronic effect of low grade-prolonged TNFα stimulation, which is more likely to reflect *in vivo* patho-physiological conditions. As expected, prolonged incubation of TNFα suppressed AICAR stimulated T172 α subunit phosphorylation and subsequent ACC phosphorylation, indicating that prolonged TNFα desensitized AMPK activation. Additionally, we demonstrated that under these conditions Fyn was activated and AMPK was phosphorylated on Y436, suggesting that this desensitization directly results from the Fyn-dependent tyrosine phosphorylation of the AMPK α subunit. Moreover we found that Fyn also regulated AICAR-dependent AMPK activation under prolonged TNFα stimulation.

Recent studies have implicated that AMPK regulates autophagy through a direct AMPK-dependent phosphorylation of ULK1 [12, 13] therefore we assessed this as a relevant physiological output. Our data further demonstrate that Fyn-dependent suppression of AMPK activity results in the inhibition of ULK1 phosphorylation and subsequent activation of autophagy, indicated by a induction in the formation of LC3-II, increase in the LC3-II/LC-I ratio, decreased p62 expression, GFP-LC3 puncta formation and autophagic flow.

Since autophagy induction by AMPK activation rescues cells from cell death [11], we examined PARP cleavage and demonstrated that TNFα induction of apoptosis is inhibited by AICAR. More importantly when Fyn was knockdown, apoptosis was completely blocked indicating that AMPK regulates TNFα-induced apoptosis and that Fyn plays a significant role in this process. However, since autophagic activity was not affected in Fyn knockdown cells and apoptosis was not induced suggest the presence of alternative pathways autophagy regulating apoptosis by Fyn.

In summary, we found that Fyn directly phosphorylates the α subunit of AMPK on Y436 and this phosphorylation suppresses AMPK activity, subsequently resulting in increased ACC and ULK1 phosphorylation. Prolonged exposure to pro-inflammatory cytokines such as TNFα decreased AICAR-dependent AMPK activation through activation of Fyn. These modulatory events manifest in the inhibition of AMPK-dependent autophagy activation and subsequent apoptosis, suggesting that targeting Fyn activity may have positive therapeutic potential as a novel pathway to activate AMPK.

## EXPERIMENTAL PROCEDURES

### Antibodies and reagents

Flag antibody was from Sigma-Aldrich (St. Louis, MO, USA), the 4G10 and GST antibodies were from Millipore (Bilerica, MA, USA), GAPDH and V5 antibodies were purchased from MBL (Woods Hole, MA, USA), Fyn antibody was from Santa Cruz Biotechnology (Santa Cruz, CA, USA), LC3 antibody was from Novus Biologicals (Littleton, CO, USA) and p62 antibody was from Enzo Life sciences (Exeter, UK). All other antibodies were purchased from Cell Signaling (Boston, MA, USA). Bafilomycin A1 and AICAR were from Sigma-Aldrich (St. Louis, MO, USA). TNFα and Metformin were from EMD Millipore (Bilerica, MA, USA), Toronto Research Chemicals (Toronto, Ontario, Canada) and Cayman Chemical (Ann Arbor, MI, USA) respectively. Leupeptin Hemisulfate was from Fisher Scientific (Pittsburgh, PA, USA). DharmaFECT Duo Transfection Reagent and siRNAs for AMPKα1, α2 and Fyn were purchased from GE Healthcare (Little Chalfont, UK). X-tremeGENE HP DNA Transfection Reagent were from Roche (Basel, Switzerland). All other reagents were purchased from Sigma-Aldrich.

### cDNA constructs

The pCMV6 Entry Myc-DDK tagged ORF clone of Homo sapiens AMPK α2 construct was obtained from Origene (Rockville, MD, USA) and used to generate the pCMV6-3Myc-2-AMPKα2-Myc DDK construct. The gene encoding AMPK α2 was amplified with the oligonucleotides: 5′- GAATTCGCCCGGGCGGGATCCCCCG GGCTGCAGATGGCTGAGAAGCAGAAGCA-3′ and 5′- AAGCTTGGTACCGGGCCCCCCCTCGAGGTCGA CGGTATCGATAACCTTATCGTCGTCATCCT-3′ and cloned to pCR-BluntII-TOPO (Invitrogen, CA, USA). This construct and pCMV-3Myc-2 (Agilent Technologies, Santa Clara, CA, USA) were digested with EcoRI and HindIII to obtain pCMV-3Myc-2-AMPKα2-Myc-DDK. GST-AMPKα1(r), pcDNA3, pcDNA3-Fyn-CA, pcDNA3-LKB1 and eGFP-hLC3 construct were constructed or obtained before [21]. Human Y436 of AMPK α2 subunit is equivalent to rat Y430 of AMPKα1. Y430F of GST-AMPKα1 was obtained by QuickChange 2-XL Site-Directed Mutagenesis Kit (Agilent Technologies, Santa Clara, CA, USA) with the pair of oligonucleotides: 5′- GGAAGGT TGTAAACCCCTTTTATTTGCGTGTGCGAAG-3 and 5′- CTTCGCAC ACGCAAATAAAAGGGGTTTACAACCTTCC-3′

### Animals

Mice over expressing the murine FynB and FynT cDNA were established as previously described [27]. pp59*^fyn^* knockout mice and their controls were obtained from The Jackson Laboratory (Bar Harbor, ME, USA). Animals were housed in a facility equipped with a 12 h light/dark cycle and fed ad libitum a standard chow diet (Research Diets, New Brunswick, NJ) containing 67% (Kcal) carbohydrates, 19% protein, and 4% fat. All studies were approved by and performed in compliance with the guidelines of the Albert Einstein College of Medicine Institutional Animal Care and Use Committee (IACUC).

### Cell culture

HEK293T, NIH3T3 and HeLa cells (ATCC, Manassas, VA, USA) were cultured in Dulbecco's Modified Eagle's Medium (Invitrogen, CA, USA) with 10% Fetal Bovine Serum (Atlanta Biologicals, Flowery Branch, GA, USA) and 1% Penicillin-Streptomycin (Life technologies, Norwalk, CT, USA). Cells were incubated at 37°C moisturized incubator with 5% CO_2_. DNA Transfection was performed using X-tremeGENE HP DNA Transfection Reagent and siRNA transfection was performed using DharmaFECT Duo Transfection Reagent according to the manufacture's instructions. siGENOME Human AMPK α1 and α2 and FYN siRNA SMART pool and Non-target siRNA were from GE Healthcare. We saw the effect of AICAR mediating AMPK phosphorylation and activity was fully regulated under confluent condition in HEK293T cells, so every experiments with HEK293T cells were performed under confluent cells condition.

### Western blot analysis

Tissues and cell extracts were prepared as described before [27]. Briefly, homogenates were centrifuged for 30 min at 14,000 rpm at 4°C, and supernatants were collected. Protein concentration was determined with the BCA^TM^ Protein Assay (Thermo Scientific, Rockford, IL). Protein lysates (40 μg) were separated on 8 to 15% reducing polyacrylamide gels and transferred onto Immobilon-P polyvinylidene difluoride membranes. Immunoblots were blocked with 5% milk in Tris-buffered saline or Odyssey Bocking Buffer (Li-COR Biotechnology, Lincoln, NE) for 60 min at room temperature and incubated overnight at 4°C with the indicated antibodies in Tris-buffered saline and 0.05% Tween 20 (TBST) containing 1% BSA. Blots were washed in TBST and incubated with horseradish peroxidase-conjugated secondary antibodies (1:30,000) for 30 min at room temperature. Membranes were washed in TBST, and antigen-antibody complexes were visualized by chemiluminescence using an ECL kit (Pierce). Alternatively, immunoblots were incubated with IRDye800CW Goat Anti Mouse (H+L) or IRDye680 Goat Anti Rabbit (H+L) secondary antibodies and signal was detected with the Odyssey Infrared Imaging System (Li-COR Biotechnology).

### Immunoprecipitation

Protein from cells and tissues were prepared in a buffer containing 50mM Tris (pH 7.4), 1% glycerol and 1% NP40 supplemented with protease inhibitor (Complete mini, Roche Pharmaceuticals, Nutley, NJ). Homogenates were centrifuged for 30 min at 14,000 rpm at 4°C, and supernatants were collected. Protein concentration was determined with the BCA^TM^ Protein Assay. Lysates (5-6 mg) were immunoprecipitated with indicated antibodies followed by incubation with ProteinA/G plus. Samples were washed 3 times and boiled and separated onto 10% SDS-PAGE followed by an overnight transfer. Western blot analysis was performed with the indicated antibodies. Alternatively, immunoprecipitation was performed with Catch and Release^®^ v2.0 Reversible Immunoprecipitation System (EMD Millipore) according to manufacturer's protocol, followed by immunoblotting with the indicated antibodies.

### ***In vitro*** AMPK phosphorylation assay

AMPKα2 subunit fusion protein and FynT was purchased from Abnova and BPS bioscience, respectively. AMPKα2 protein (1 μg) was incubated with the recombinant FynT kinase active (1.8U) in presence of ATP and kinase buffer (Cell Signaling) and kinase reaction was performed for 1 h at 35°C. Samples were separated on 10% SDS-polyacrylamide gels and immunoblotting was performed with 4G10 monoclonal antibody.

### Fyn activity assay

Fyn activity assay was performed with Universal Tyrosine Kinase Assay kit (Takara Bio Inc, Shiga, Japan) as described previously [27]. Briefly, homogenates were prepared and protein concentration was determined by BCA method. Samples (0.5-1 mg) were incubated with 4 μg of Fyn rabbit polyclonal antibody (Santa Cruz Biotechnology) coupled with Catch and Release^®^ v2.0 Reversible Immunoprecipitation System (EMD Millipore). Samples were washed three times with NP-40 lysis buffer and once with the kinase reaction buffer. Immuno-complexes were diluted 2.5 fold and 40 μl samples and 10 μl ATP solution were incubated in an ELISA plate provided. Measurement of the kinase activity was performed according to the manufacturer's protocol.

### Immunofuorescence

HEK293T cells were transfected with GFP-human LC3 construct. Transfected cells were washed with PBS and fixed for 10 min in PBS containing 4% PFA. Cells were imaged using a confocal fluorescence microscope (FV10i; Olympus).

### Statistics

All data are presented as mean ± s.e.m. Significant data were defined as *p* < 0.05 using Student's *t* test or ANOVA.

## SUPPLEMENTARY MATERIALS FIGURES



## References

[1] Yamada E, Singh R (2012). Mapping autophagy on to your metabolic radar. Diabetes.

[2] Altman BJ, Rathmell JC (2012). Metabolic stress in autophagy and cell death pathways. Cold Spring Harbor perspectives in biology.

[3] Singh R, Kaushik S, Wang Y, Xiang Y, Novak I, Komatsu M, Tanaka K, Cuervo AM, Czaja MJ (2009). Autophagy regulates lipid metabolism. Nature.

[4] Auranen M, Villanova M, Muntoni F, Fardeau M, Scherer SW, Kalino H, Minassian BA (2000). X-linked vacuolar myopathies: two separate loci and refined genetic mapping. Annals of neurology.

[5] Raben N, Schreiner C, Baum R, Takikita S, Xu S, Xie T, Myerowitz R, Komatsu M, Van der Meulen JH, Nagaraju K, Ralston E, Plotz PH (2010). Suppression of autophagy permits successful enzyme replacement therapy in a lysosomal storage disorder--murine Pompe disease. Autophagy.

[6] Singh R, Xiang Y, Wang Y, Baikati K, Cuervo AM, Luu YK, Tang Y, Pessin JE, Schwartz GJ, Czaja MJ (2009). Autophagy regulates adipose mass and differentiation in mice. The Journal of clinical investigation.

[7] Zhang Y, Goldman S, Baerga R, Zhao Y, Komatsu M, Jin S (2009). Adipose-specific deletion of autophagy-related gene 7 (atg7) in mice reveals a role in adipogenesis. Proceedings of the National Academy of Sciences of the United States of America.

[8] Ebato C, Uchida T, Arakawa M, Komatsu M, Ueno T, Komiya K, Azuma K, Hirose T, Tanaka K, Kominami E, Kawamori R, Fujitani Y, Watada H (2008). Autophagy is important in islet homeostasis and compensatory increase of beta cell mass in response to high-fat diet. Cell metabolism.

[9] Jung HS, Chung KW, Won Kim J, Kim J, Komatsu M, Tanaka K, Nguyen YH, Kang TM, Yoon KH, Kim JW, Jeong YT, Han MS, Lee MK (2008). Loss of autophagy diminishes pancreatic beta cell mass and function with resultant hyperglycemia. Cell metabolism.

[10] Lapaquette P, Guzzo J, Bretillon L, Bringer MA (2015). Cellular and Molecular Connections between Autophagy and Inflammation. Mediators of inflammation.

[11] Law BY, Mok SW, Chan WK, Xu SW, Wu AG, Yao XJ, Wang JR, Liu L, Wong VK (2016). Hernandezine, a novel AMPK activator induces autophagic cell death in drug-resistant cancers. Oncotarget.

[12] Kim J, Kundu M, Viollet B, Guan KL (2011). AMPK and mTOR regulate autophagy through direct phosphorylation of Ulk1. Nature cell biology.

[13] Egan DF, Shackelford DB, Mihaylova MM, Gelino S, Kohnz RA, Mair W, Vasquez DS, Joshi A, Gwinn DM, Taylor R, Asara JM, Fitzpatrick J, Dillin A (2011). Phosphorylation of ULK1 (hATG1) by AMP-activated protein kinase connects energy sensing to mitophagy. Science.

[14] Crute BE, Seefeld K, Gamble J, Kemp BE, Witters LA (1998). Functional domains of the alpha1 catalytic subunit of the AMP-activated protein kinase. The Journal of biological chemistry.

[15] Hardie DG, Ross FA, Hawley SA (2012). AMPK: a nutrient and energy sensor that maintains energy homeostasis. Nature reviews Molecular cell biology.

[16] Woods A, Cheung PC, Smith FC, Davison MD, Scott J, Beri RK, Carling D (1996). Characterization of AMP-activated protein kinase beta and gamma subunits. Assembly of the heterotrimeric complex in vitro. The Journal of biological chemistry.

[17] Hawley SA, Pan DA, Mustard KJ, Ross L, Bain J, Edelman AM, Frenguelli BG, Hardie DG (2005). Calmodulin-dependent protein kinase kinase-beta is an alternative upstream kinase for AMP-activated protein kinase. Cell metabolism.

[18] Woods A, Johnstone SR, Dickerson K, Leiper FC, Fryer LG, Neumann D, Schlattner U, Wallimann T, Carlson M, Carling D (2003). LKB1 is the upstream kinase in the AMP-activated protein kinase cascade. Current biology : CB.

[19] Kajita K, Mune T, Ikeda T, Matsumoto M, Uno Y, Sugiyama C, Matsubara K, Morita H, Takemura M, Seishima M, Takeda J, Ishizuka T (2008). Effect of fasting on PPARgamma and AMPK activity in adipocytes. Diabetes research and clinical practice.

[20] Bastie CC, Zong H, Xu J, Busa B, Judex S, Kurland IJ, Pessin JE (2007). Integrative metabolic regulation of peripheral tissue fatty acid oxidation by the SRC kinase family member Fyn. Cell metabolism.

[21] Yamada E, Pessin JE, Kurland IJ, Schwartz GJ, Bastie CC (2010). Fyn-dependent regulation of energy expenditure and body weight is mediated by tyrosine phosphorylation of LKB1. Cell metabolism.

[22] Zhang S, Qi Q, Chan CB, Zhou W, Chen J, Luo HR, Appin C, Brat DJ, Ye K (2016). Fyn-phosphorylated PIKE-A binds and inhibits AMPK signaling, blocking its tumor suppressive activity. Cell death and differentiation.

[23] Salmond RJ, Filby A, Qureshi I, Caserta S, Zamoyska R (2009). T-cell receptor proximal signaling via the Src-family kinases, Lck and Fyn, influences T-cell activation, differentiation, and tolerance. Immunological reviews.

[24] Palacios EH, Weiss A (2004). Function of the Src-family kinases, Lck and Fyn, in T-cell development and activation. Oncogene.

[25] Saito YD, Jensen AR, Salgia R, Posadas EM (2010). Fyn: a novel molecular target in cancer. Cancer.

[26] Davidson D, Viallet J, Veillette A (1994). Unique catalytic properties dictate the enhanced function of p59fynT, the hemopoietic cell-specific isoform of the Fyn tyrosine protein kinase, in T cells. Molecular and cellular biology.

[27] Yamada E, Bastie CC, Koga H, Wang Y, Cuervo AM, Pessin JE (2012). Mouse skeletal muscle fiber-type-specific macroautophagy and muscle wasting are regulated by a Fyn/STAT3/Vps34 signaling pathway. Cell reports.

[28] Steinberg GR, Kemp BE (2009). AMPK in Health and Disease. Physiological reviews.

[29] Harris J (2011). Autophagy and cytokines. Cytokine.

[30] Steinberg GR, Michell BJ, van Denderen BJ, Watt MJ, Carey AL, Fam BC, Andrikopoulos S, Proietto J, Görgün CZ, Carling D, Hotamisligil GS, Febbraio MA, Kay TW (2006). Tumor necrosis factor alpha-induced skeletal muscle insulin resistance involves suppression of AMP-kinase signaling. Cell metabolism.

[31] Tang XX, Chen H, Yu S, Zhang L, Caplan MJ, Chan HC (2010). Lymphocytes accelerate epithelial tight junction assembly: role of AMP-activated protein kinase (AMPK). PloS one.

[32] Hong SW, Lee J, Park SE, Rhee EJ, Park CY, Oh KW, Park SW, Lee WY (2014). Activation of AMP-Activated Protein Kinase Attenuates Tumor Necrosis Factor-alpha-Induced Lipolysis via Protection of Perilipin in 3T3-L1 Adipocytes. Endocrinol Metab (Seoul).

[33] Bollheimer LC, Buettner R, Pongratz G, Brunner-Ploss R, Hechtl C, Banas M, Singler K, Hamer OW, Stroszczynski C, Sieber CC, Fellner C (2012). Sarcopenia in the aging high-fat fed rat: a pilot study for modeling sarcopenic obesity in rodents. Biogerontology.

[34] Corton JM, Gillespie JG, Hawley SA, Hardie DG (1995). 5-aminoimidazole-4-carboxamide ribonucleoside. A specific method for activating AMP-activated protein kinase in intact cells?. European journal of biochemistry / FEBS.

[35] Grahame Hardie D (2014). AMP-activated protein kinase: a key regulator of energy balance with many roles in human disease. Journal of internal medicine.

[36] Mihaylova MM, Shaw RJ (2011). The AMPK signalling pathway coordinates cell growth, autophagy and metabolism. Nature cell biology.

[37] Snima KS, Pillai P, Cherian AM, Nair SV, Lakshmanan VK (2014). Anti-diabetic drug metformin: challenges and perspectives for cancer therapy. Current cancer drug targets.

[38] Davies SP, Helps NR, Cohen PT, Hardie DG (1995). 5′-AMP inhibits dephosphorylation, as well as promoting phosphorylation, of the AMP-activated protein kinase. Studies using bacterially expressed human protein phosphatase-2C alpha and native bovine protein phosphatase-2AC. FEBS letters.

[39] Marley AE, Sullivan JE, Carling D, Abbott WM, Smith GJ, Taylor IW, Carey F, Beri RK (1996). Biochemical characterization and deletion analysis of recombinant human protein phosphatase 2C alpha. The Biochemical journal.

[40] Jensen TE, Rose AJ, Jørgensen SB, Brandt N, Schjerling P, Wojtaszewski JF, Richter EA (2007). Possible CaMKK-dependent regulation of AMPK phosphorylation and glucose uptake at the onset of mild tetanic skeletal muscle contraction. American journal of physiology Endocrinology and metabolism.

[41] Anderson KA, Means RL, Huang QH, Kemp BE, Goldstein EG, Selbert MA, Edelman AM, Fremeau RT, Means AR (1998). Components of a calmodulin-dependent protein kinase cascade. Molecular cloning, functional characterization and cellular localization of Ca2+/calmodulin-dependent protein kinase kinase beta. The Journal of biological chemistry.

[42] Li X, Wang L, Zhou XE, Ke J, de Waal PW, Gu X, Tan MH, Wang D, Wu D, Xu HE, Melcher K (2015). Structural basis of AMPK regulation by adenine nucleotides and glycogen. Cell research.

