# Supplementary material for: Fyn phosphorylates AMPK to inhibit AMPK activity and AMP-dependent activation of autophagy
Source: Oncotarget. 2016 Sep 8;7(46):74612–29. doi: 10.18632/oncotarget.11916 (PMC5340180; doi:10.18632/oncotarget.11916)
Supplement: Supplementary file 1 [file oncotarget-07-74612-s001.pdf]

Fyn phosphorylates AMPK to inhibit AMPK activity and AMP-dependent activation of autophagy

Supplementary Material

A

Protein Name	Site	Peptide	HSA-FynT #1	HSA-FynT #2	Fyn KO #1	Fyn KO #2
STAT3	Y705	YCRPESQEHPEADPGSAAPY*LK	27,205	33,603	10,586	8,363
AMPK1	Y436	VVNPY*YLR	72,612	56,441	4,437	5,259

B

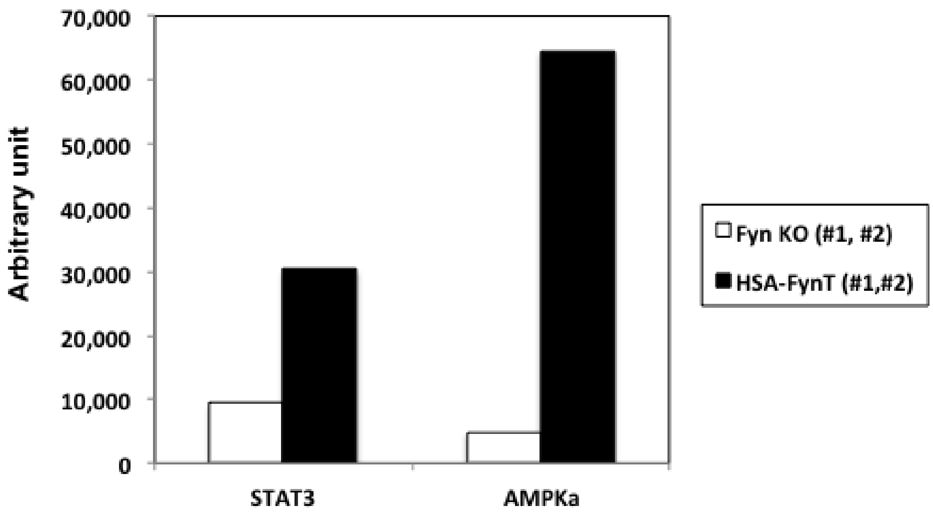

Supplementary Figure 1.

Cell Signaling Technology (Boston, MA, USA)'s PhosphoScan® proteomics platform was used to identify and quantify differences in phosphorylation in two FynT transgenic mice and FynKO mice muscle tissue. The PhosphoScan method combines the isolation of phosphorylated peptides from protease digested protein extracts using CST's proprietary immunoaffinity purification method with the identification and quantitation of peptides by liquid chromatography, tandem mass spectrometry (LC-MS/MS). Phosphorylation site and fold increase in FynT transgenic mice compared to FynKO mice are showed in supplementary Fig 1A and B.

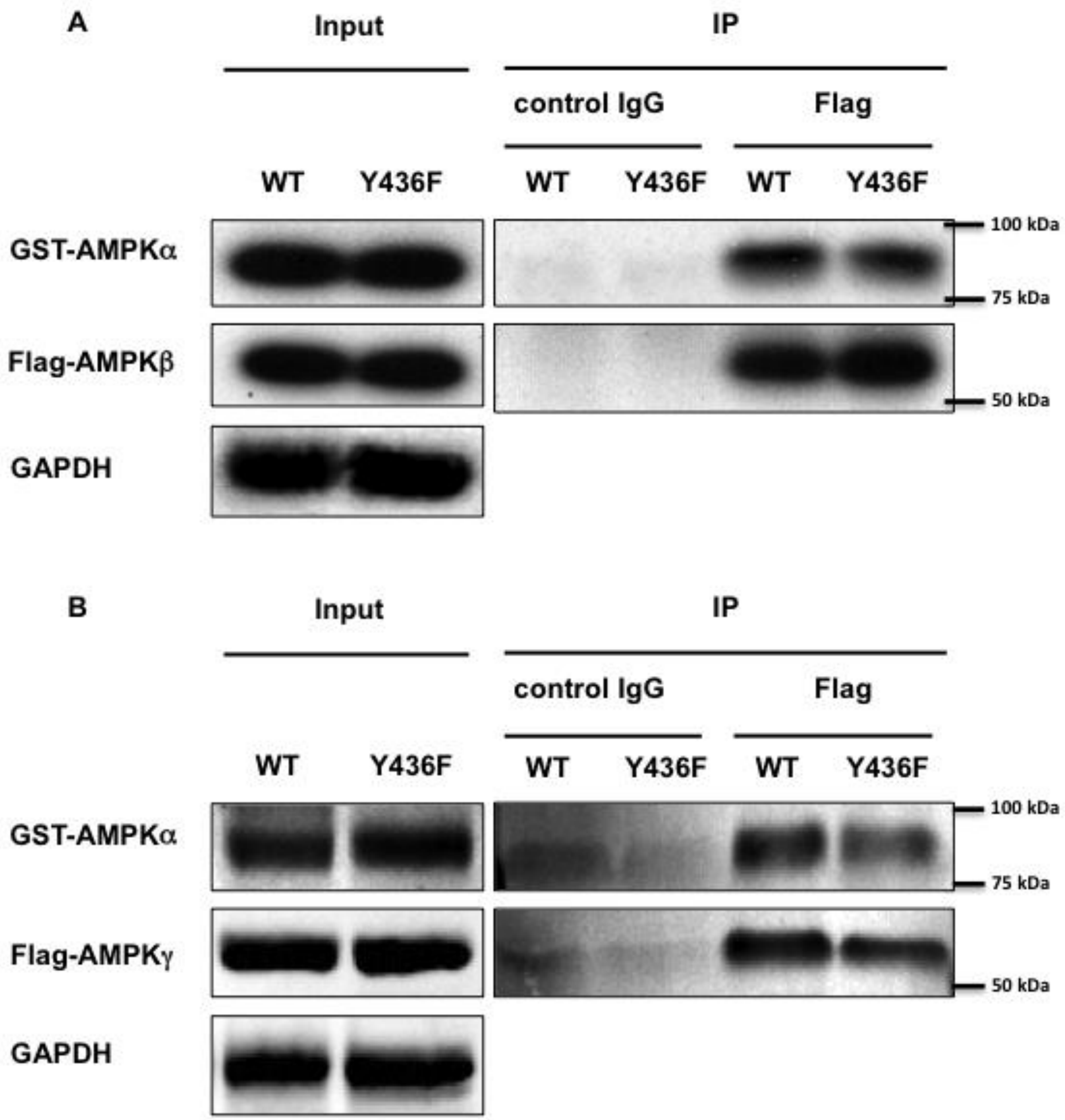

Supplementary Figure 2

(A) HEK293T cells were co-transfected by either GST-AMPKα WT or Y436F with Flag-AMPKβ2 subunit. Immunoprecipitation was performed using a Flag antibody. Immunoblots were performed with the indicated antibodies. (B) HEK293T cells were co-transfected by either GST-AMPKα WT or Y436F with Flag-AMPKγ3 subunit. Immunoprecipitation was performed with a Flag antibody. Immunoblots were performed with the indicated antibodies.

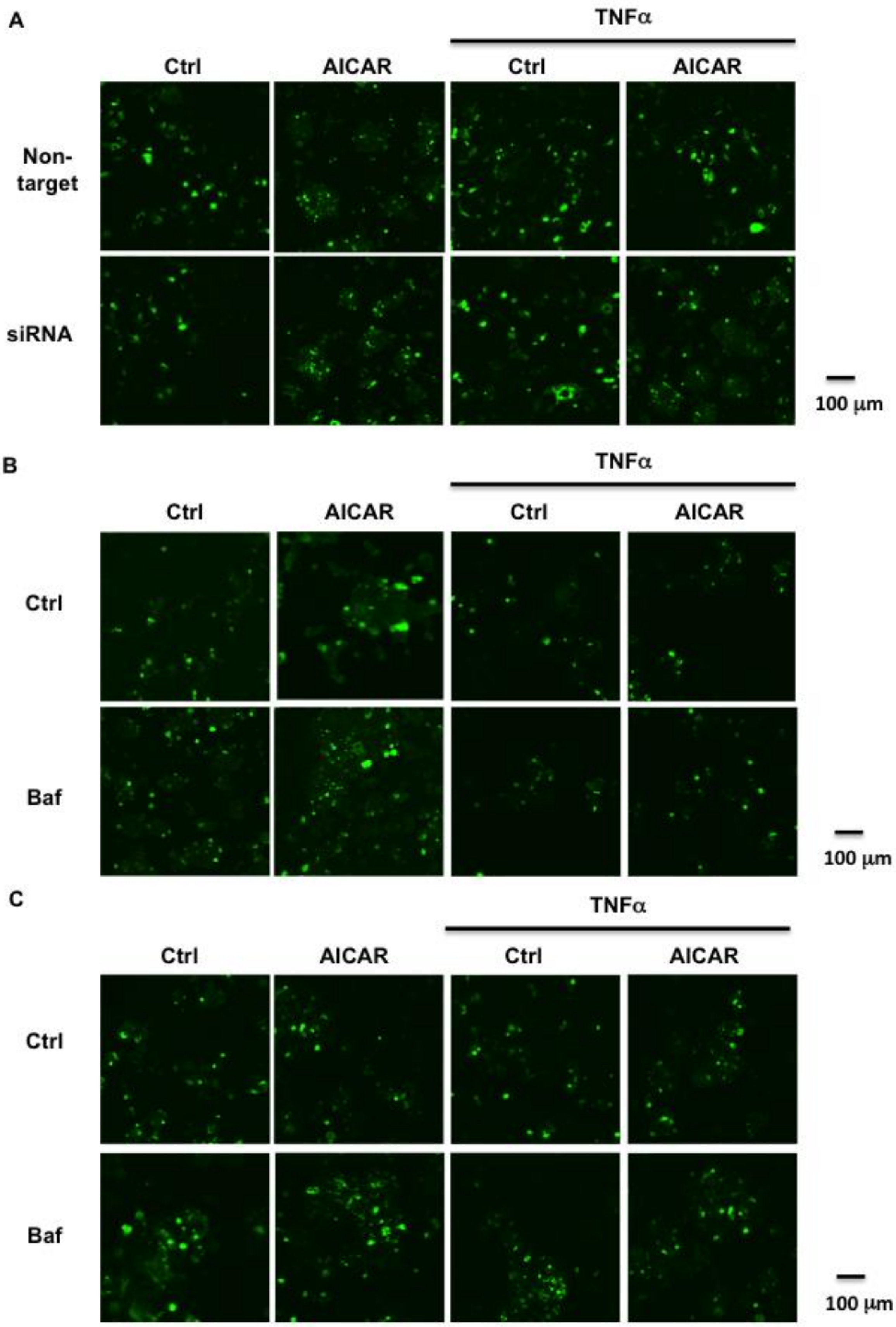
A
TNFα
Ctrl
AICAR
Ctrl
AICAR
Non-target
siRNA
100 μm
B
TNFα
Ctrl
AICAR
Ctrl
AICAR
Ctrl
Baf
100 μm
C
TNFα
Ctrl
AICAR
Ctrl
AICAR
Ctrl
Baf
100 μm

Supplementary Figure 3

(A) HEK293T cells were transfected with both siRNA for Fyn and GFP-human LC3 construct. Cells were treated with 10 ng/ml TNFα for 36 h with or without 2 mM AICAR for 10 min. Cells were fixed with 4% paraformaldehyde and mounted with DAKOCytomation Fluorescent Mounting Medium (S3023). These are representative images of low power field from experiments independently performed 3 times. (B) HEK293T cells were transfected with both siRNA for Fyn and GFP-human LC3 construct. Cells were treated with 10 ng/ml TNFα for 36 h with or without 100 nM Bafilomycin for 4 h and 2 mM AICAR for 10 min. Cells were fixed with 4% paraformaldehyde and mounted with DAKOCytomation Fluorescent Mounting Medium (S3023). These are representative images of low power field from experiments of non-target siRNA transfected cells (B) or Fyn siRNA transfected cells (C) independently performed 3 times.
